# Relationship between Biofilm Formation and Antibiotic Resistance and Adherence Genes in *Staphylococcus aureus* Strains Isolated from Raw Cow Milk in Shahrekord, Iran

**DOI:** 10.1155/2022/6435774

**Published:** 2022-10-25

**Authors:** Rasul Pajohesh, Elahe Tajbakhsh, Hassan Momtaz, Ebrahim Rahimi

**Affiliations:** ^1^Department of Microbiology, Faculty of Basic Sciences, Shahrekord Branch, Islamic Azad University, Shahrekord, Iran; ^2^Department of Food Hygiene, Faculty of Veterinary Medicine, Shahrekord Branch, Islamic Azad University, Shahrekord, Iran

## Abstract

The production of biofilms by *S. aureus* contributes significantly to treatment failures. The present study aims to establish the relationship between biofilm formation and antibiotic resistance and adhesion genes in *Staphylococcus aureus* strains isolated from raw cow milk in Shahrekord, Iran. A total of 90 samples of raw cow's milk were collected. Presumptive *S. aureus* strains were obtained using Baird-Parker plates after enrichment in tryptone soy broth, and final colonies were selected from brain heart infusion. Additional tests such as coagulase were done, and the identification was confirmed by the detection of the *aroA* gene. Biofilm producing strains were screened using a spectrophotometry method applied to microplates. Crystal violet staining was used to quantify the formation of biofilm. An antibiotic susceptibility test was performed using the Kirby–Bauer disc diffusion method. PCR was used to detect several biofilm and antibiotics resistance related genes. The chi-square test and Fisher's exact test were used to establish a statistically significant relationship between biofilm reaction and antibiotic resistance (*p* value <0.05). Results show a moderate (38.88%) recovery rate of *S. aureus* in milk and 65.71% of the isolates were strong biofilm producers. Antibiotic susceptibility tests show an alarming rate of resistance to beta-lactam antibiotics, especially penicillin (100%), ampicillin (91.42%), and oxacillin (71.42%). This finding correlates with antibiotic resistance gene detection, in which the gene *blaZ* was most found (71.42%), followed by *mecA* and *Aac-D* (42.85%). Detection of biofilm-related genes shows that all the genes targeted were found among *S. aureus* isolates. Statistical tests show a significant correlation between biofilm production and antibiotic resistance in *S. aureus*. This study revealed that there is a significant correlation between biofilm production and antibiotic resistance in *S. aureus* isolated from raw milk. These results highlight the need for regular surveillance of the occurrence of *S. aureus* strains in milk and milk products in Iran.

## 1. Introduction

*Staphylococcus aureus* is a Gram-positive facultatively anaerobic cocci [1, 2]. *Staphylococcus aureus* is well known to cause zoonotic diseases and is one of the main agents of food poisoning [3, 4]. Indeed, its presence in food represents a serious health problem as it can produce a wide range of virulence factors such as enzymes and exotoxins that cause food poisoning [5]. Handling, close contact, and consumption of animal products are the main modes of contamination of these bacteria [6].

Among the wide variety of animal products that *S. aureus* can contaminate, milk is an ideal substrate for its growth and for the production of staphylococcal toxins that affect the quality of the milk [7]. Contamination of raw milk with *S. aureus* can occur from animal skin, mucosal surfaces, infected glands, milking equipment, milker's hands, and the environment [8].

In addition, the pasteurisation step does not inhibit the activity of staphylococcal enterotoxins, as they can be very heat-resistant [9]. Many studies have reported the presence of *S. aureus* strains producing staphylococcal toxins in milk across the world [10]. Consumption of milk poisoned with staphylococcal enterotoxins can cause nausea, vomiting, and abdominal cramps [11].

Apart from the production of enterotoxin, the ability of *S. aureus* to form a biofilm is essential for its long existence in a harsh environment [12, 13]. Biofilms are structured clusters of bacterial cells embedded in a polymeric matrix and attached to a surface [14]. Biofilm formation in *S. aureus* is not a simple process and is encoded by many genes, such as *rbf* [15], *mgrA* [16], and *icaR* [17]. Biofilm formation defends bacteria against desiccation, the host's immune defences, and the action of oxidising biocides and antibiotics [18]. *S. aureus* strains can be resistant to one or more antibiotics and can cause serious and difficult-to-treat infections [11, 19].

As many pathogenic bacteria produce biofilms, there is growing interest in studying the correlation between biofilm production and antimicrobial susceptibility profile [20]. Thus, the role of biofilm has been studied on *S. aureus* strains isolated from humans [21], pork [22], dairy products [23], cows [24], and milk [25]. The study of multidrug-resistant *S. aureus* in dairy production is of great concern as it has a negative impact on milk production and may represent a public health problem for workers involved in food production [26].

Dairy production is one of the main high-income sectors in the world [6]. In Iran, the dairy sector is one of the main traditional and economic activities, and milk production has increased to a level of about 9 billion kg of milk per year [27]. With the high demand, the sale of raw milk for direct consumption may have increased human exposure to zoonotic agents [28]. Numerous studies conducted in Iran recovered *S. aureus* from dairy products [1, 29–31]. However, data on the role of biofilm formation of *S. aureus* recovered from dairy products in Iran and the antimicrobial susceptibility profile are scarce. This information is important to better understand the evolution of *S. aureus* and to assess the risk to those involved in dairy production. In this regard, the present study aims to establish the relationship between biofilm formation and antibiotic resistance and adhesion genes in *Staphylococcus aureus* strains isolated from raw cow milk in Shahrekord, Iran.

## 2. Materials and Methods

### 2.1. Sampling

In this cross-sectional study, a total of 90 raw cow milk samples (Holstein Friesian) were collected randomly from May to November 2019 in Shahrekord, Iran. Samples were randomly collected from various herds through Shahrekord. The herds were selected by convenience (i.e., the owners of the herds agreed to participate in the study), as all invited herd owners had an existing relationship with the research team. The animals from whose milk samples were collected for this study were clinically healthy and the milk samples showed normal physical characteristics. The cows in each herd that have shown obvious changes in milk, heat or udder swelling, and/or heat and mammary gland swelling (i.e., clinical mastitis) were not selected. Samples were collected under sterile hygienic conditions according to the International Dairy Federation guidelines and were immediately transported to the microbiology and biotechnology laboratories of Islamic Azad University, Shahrekord Branch, Iran [32].

### 2.2. Bacterial Isolation

Isolation of *S. aureus* was performed following the method described by Cenci-Goga et al. [33]. The first isolation medium was tryptose blood agar base containing washed bovine red blood cells (HIMEDIA); 1 ml of milk was spread on this medium and incubated at 37°C for 48 h. Creamy grayish white or golden yellow colonies 3 to 5 mm in diameter with distinct zones of hemolysis were considered presumptive *S. aureus* colonies. The tests performed to identify *the S. aureus* isolates included growth characteristics on blood agar, Gram staining, catalase test, growth on Mannitol salt agar base, slide and tube coagulase tests, and the presence of black clony on Bird-Parker agar.

### 2.3. Biofilm Formation

*S. aureus* ATCC25923 (biofilm-forming) and *S. epidermidis* ATCC12228 (not biofilm-forming) were respectively used as positive and negative controls. As specified by Pajohesh et al. [34], spectrophotometry was applied in microplates using crystal violet staining to quantify the formation of biofilm. For this purpose, a mixture was reached by adding 20 ml of bacterial log phase culture to 200 ml of fresh 1% glucose BHI using 96-well flat-bottom microtiter plates. BHI without bacteria was used as empty. The plates were put for incubation at 37°C for 48 hr. Using aerobic conditions after each sampling, 300 mL of sterile phosphate-buffered saline was used to wash the wells three times; then, they were inverted for drainage. After that, 200 mL of methanol was added to each well, and the plates were dried for 15 minutes. 150 mL of 0.1% crystalline violet solution was used for staining of sticky cells for 15 minutes and then sterile water was used twice to wash. 150 mL of 95% ethanol was used for 10 minutes to dissolve the purple crystal violet. The optical density of each well was measured at 570 nm (OD570) using the Multiskan FC (Thermo Fisher Scientific Inc., Madison, WI). The interpretation of the results concerning biofilm formation was made according to the following rule: (OD570 ≥ 1) as strong, (0.1 ≤ OD570 < 1) weak positive, (OD570 < 0.1) as negative. *S. aureus* (ATCC 25923), and *S. epidermidis* (ATCC 12228) were applied as positive and negative controls, respectively.

### 2.4. Antibiotics Susceptibility Test

The Kirby-Bauer disc diffusion method was applied by applying Mueller–Hinton agar (Merck), following the Clinical and Laboratory Standards Institute guidelines to carry out the antimicrobial susceptibility tests. As suggested by CLSI [35], the disc-diffusion method on Mueller–Hinton agar was applied to examine the susceptibility of all antibiotics.

The procedure is as follows; *S. aureus* isolates were put to grow during the night on blood agar. The sterile saline water equivalent to a 0.5-McFarland standard was used to achieve the colony suspension; then, 100 *µ*l of suspension was spilled over the media plate and antibiotic disc was put aseptically on the surface of the protected media plate. Next, these plates were put for incubation at 30°C for methicillin and 35 C for other antibiotics for 24 hr. The following antibiotic disks were used; beta-lactam antibiotics such as methicillin (MET 5 *µ*g), penicillin G (P 10 *µ*g), ampicillin (AMP 25 *µ*g), amoxycillin (AMS 30 *µ*g), oxacillin (OX 5 *µ*g), macrolides such as erythromycin (E 10 *µ*g), aminoglycoside antibiotics such as gentamycin (GEN 20 *μ*g), kanamycin (K 20 *µ*g), streptomycin (S 20 *µ*g), lincosamides such as: lincomycin (L 15 *µ*g), clindamycin (CC 2 *µ*g). Glycopeptide antibiotics such as vancomycin (V 10 *µ*g). Chloramphenicol (C 30 *µ*g), tetracycline (TE 30 *µ*g), and rifampicin (R 30 *µ*g).

### 2.5. DNA Extraction and Polymerase Chain Reaction (PCR)

The DNA extraction kit PrepMan® Ultra Reagent (Applied Biosystems, Woolston, Warrington, United Kingdom) was used to extract genomic DNA from *S. aureus* isolates following the manufacturer's instructions. Total DNA was determined at an optical density of 260 nm.

*S. aureus* isolates were evaluated by PCR for the presence of the *aroA* gene, as described by Dastmalchi Saei et al. [36].

The PCR was performed in a 25-*µ*l reaction mixture containing 12.5 *µ*l of 2x master mix (0.04 U/*µ*l Taq DNA polymerase, reaction buffer, 3 mM MgCl_2_ 0.4 mM of each dNTP), 0.4 *µ*M of each primer, and 2 *µ*l of template DNA. For the negative control, sterile water was added instead of nucleic acids. As a positive control, we used *S. aureus* ATCC 29213. The molecular amplification was conducted for the detection of the *aroA* gene by using species specific primers and thermal profile, which is shown in Table 1 [37–42]. Analysis of the PCR products for *aroA* was performed by agarose gel electrophoresis using a 1.2% gel and 0.5 *µ*g/ml ethidium bromide in 0.5x TBE electrophoresis buffer at 80 V for 1 h and photographed under UV light. A single PCR product 1,153-bp was obtained from all *S. aureus* DNA extracts. The size of the PCR product was determined by comparison to the ΦX174 DNA/HaeIII markers (Fermentas, Germany).

The oligonucleotide primers of the biofilm's genes encoding and antibiotic resistance genes, multiplex PCR programs, and the product size are indicated in Table 1. A DNA thermal cycler (Mastercycler Gradient, Eppendorf, Germany) was used to perform the PCR. The ethidium bromide and electrophoresed were used in 1.5% agarose gel at 80 volts for 30 minutes to stain amplifiers. UV doc gel documentation systems (Uvitec, UK) were used to visualize and photograph PCR products. A comparison was run between PCR products and 100 bp DNA markers (Fermentas *n*, Germany).

### 2.6. Statistical Analysis

The data were transferred to a Microsoft Excel spreadsheet (version 15; Microsoft Corp., Redmond, WA, USA) for analysis. Using statistical software (version 16; SPSS Inc., Chicago, USA), the chi-square test and Fisher's exact two-tailed test analysis were performed, and differences were considered significant at values of *p* < 0.05.

## 3. Results

Of the 90 milk samples, 35 samples (38.88%) were positive for *S. aureus* and all isolates were approved by PCR for the presence of the *aroA* gene (this confirms that PCR here is pretty useless).  Table 2 shows the results for the detection of biofilm formation. Biofilm formation was strongly and weakly observed in 65.71% and 20% of isolated *S. aureus* strains, respectively.

Table 3 shows the results for the antibiotic resistance pattern of *S. aureus* strains. All the isolates (100%) exhibited resistance to penicillin and almost all were resistant to ampicillin (91.42%). A high number of isolates (71.42%) exhibited resistance to oxacillin and a significant number of isolates were resistant to methicillin and kanamycin (42.85%). All isolates were sensitive to vancomycin and rifampicin.

Table 4 shows the results for the antibiotic resistance pattern based on biofilm reaction. All strong biofilm producer isolates (100%) exhibited resistance to penicillin and almost all were resistant to ampicillin and oxacillin (95.65%). A high number of strong biofilm producer isolates (65.22%) were resistant to methicillin and kanamycin. Based on Fisher's exact test, there is a statistically significant relationship between strong biofilm reaction and resistance to penicillin G, ampicillin, oxacillin, and gentamycin antibiotics (*p* value <0.05). But there is no statistically significant relationship between other antibiotics and biofilm reaction (*p* value >0.05).

Table 5 shows the results for the prevalence of antibiotic resistance genes. The gene *blaZ* was found on a high number (71.42%) of *S. aureus* isolates, and 42.85% of isolates carry the resistance genes *mecA* and *Aac-D*. The same number of isolates (28.57%) carries resistance genes *tetK* and *tetM* and 14.28% of isolates carry the genes *linA*, *ermA*, and *ermC*.

Table 6 presents the prevalence of antibiotic resistance genes in biofilm-forming and nonforming isolates. According to this table, all strong biofilm-producing strains carry the *blaZ* genes and a significant number of isolates carry *mecA* and *tetK* with a prevalence of 65.22% and 43.48%, respectively. Thus, the most prevalent genes were mainly found in strong biofilm producing strains. Also, based on the chi-square test, there is a statistically significant relationship between strong biofilm reactions and antibiotic resistance (*p* value <0.05). Based on Fisher's exact test, there is no statistically significant relationship between any antibiotic and weak and negative biofilm reactions (*p* value >0.05).

Table 7 presents the genotypic evaluation of biofilm production in *Staphylococcus aureus* isolates. All the 8 genes encoding biofilm production were detected in *Staphylococcus aureus* isolates. The minimum frequency (71.43%) was found for the gene *clfb*.

Table 8 presents the adherence of attachment factor genes in *Staphylococcus aureus* isolates based on biofilm reaction. The attachment factor genes *icaA*, *icaB*, *icaC*, *icaD,* fnbA, and fnbB were present in all strong biofilm producing strains, and a high number of these isolates (86.96%) carry *bap*, *cflA,* and *cflB*. Based on Fisher's exact test, there is a statistically significant relationship between the active genes and strong biofilm reaction (*p* value <0.05). But there is no statistically significant relationship between any of the active genes and weak or negative biofilm reactions (*p* value >0.05).

## 4. Discussion

*Staphylococcus aureus* is a common pathogenic bacterium for both humans and animals [43–45]. Its pathogenicity depends on the wide range of staphylococcal enterotoxins that it can produce and which have an adverse effect on humans and animal organisms [44, 46]. The production of a biofilm enhances its virulence as it resists substances such as antibiotics that can inhibit its growth [47]. The present study aims to establish the relationship between biofilm formation and antibiotic resistance and adhesion genes in *Staphylococcus aureus* strains isolated from raw cow milk in Shahrekord, Iran.

The results for the presence of *S. aureus* in raw cow milk show that of the 90 milk samples, 35 samples (38.88%) were positive for *S. aureus* and all isolates were approved by PCR for the presence of the *aroA* gene. Previously, several studies have found similar rates of isolation of *S. aureus* in milk [48, 49]. The presence of *S. aureus* in milk can be explained by poor hygiene conditions during production, handling, and/or distribution [23]. In addition, milk provides good growing conditions for *S. aureus*, which can survive in products for a long time. Investigation of phenotypic biofilm production showed that biofilm formation was strongly and weakly observed in 65.71% and 20% of isolated *S. aureus* strains, respectively. This result is consistent with some studies that also reported that most of the *S. aureus* strains recovered from milk were biofilm producers [50, 51]. This result confirms that the majority of *S. aureus* isolated from raw milk are biofilm producers [52]. According to Shen et al., the presence of milk may play an important role in biofilm production by *S. aureus* [52]. The sugar (glucose) content of the milk positively influenced biofilm formation [53].

Antibiotic resistance pattern results show that all the isolates (100%) exhibited resistance to penicillin, and almost all were resistant to ampicillin (91.42%). This alarming rate of resistance to beta-lactam antibiotics is corroborated by some recent findings [54, 55] and can be explained by the common use of *β*-lactams in the treatment of bovine mastitis [56]. A study carried out in Kenya by Mbindyo et al. found that 71.4% of *S. aureus* strains exhibited resistance to ampicillin [57]. Another study reported that the frequency of resistance to penicillin G was 85.2% [58]. A high number of isolates (71.42%) exhibited resistance to oxacillin and a significant number of isolates were resistant to methicillin and kanamycin (42.85%). This pattern of multidrug resistance, particularly methicillin resistance, is increasingly being reported worldwide [23, 59, 60]. The emergence of resistance observed is associated with the misuse and overuse of antibiotics in farming [61]. In many developing countries, such as Iran, most of these antibiotics are cheap and easy to find and do not require a veterinary prescription to purchase [62].

The results for antibiotic resistance pattern based on biofilm reaction show that all strong biofilm producer isolates (100%) exhibited resistance to penicillin and almost all were resistant to ampicillin and oxacillin (95.65%). A high number of strong biofilm producer isolates (65.22%) were resistant to methicillin and kanamycin. Similar results were found by Manandhar et al. [63], who reported a high frequency of multiple antibiotics resistance, such as penicillin, cefoxitin, tetracycline, clindamycin, and chloramphenicol, from clinical isolates.

Based on Fisher's exact test, there is a statistically significant relationship between strong biofilm reaction and resistance to penicillin G, ampicillin, oxacillin, and gentamycin antibiotics (*p* value <0.05). This finding is not in line with the results reported by [63, 64], who did not find any significant difference in biofilm production between methicillin-resistant *S. aureus* and methicillin-sensitive *S. aureus*. Other studies found that biofilm production correlated well with methicillin resistance [24]. The discrepancies in the findings can be explained by differences in the interpretation of results [25]. Indeed, various methods, such as the Congo red plate assay [65], crystal violet (CV) assay [66], and microtitre plate assay [34], can be used to screen biofilm-producing strains.

The results for the prevalence of antibiotic resistance genes show that the gene *blaZ* was the most prevalent (71.42%), followed by *mecA* and *Aac-D* (42.85%). These results are in line with the observed antibiotic pattern with a high number of resistances to beta-lactam antibiotics (penicillin, ampicillin, oxacillin, and methicillin) certainly due to the gene *blaZ*. It highlights a relationship between *blaZ* and resistance to beta-lactams. Similar results have been reported [67, 68]. The significant number of isolates that carry *mecA* can explain the high rate of resistance to methicillin and multiple antibiotics [25].

The prevalence of antibiotic resistance genes in biofilm-forming and nonforming isolates results show that the most prevalent genes were mainly found in strong biofilm-producing strains. Also, based on the chi-square test, there is a statistically significant relationship between strong biofilm reactions and antibiotic resistance (*p* value <0.05). Based on Fisher's exact test, there is no statistically significant relationship between any antibiotic and weak and negative biofilm reactions (*p* value >0.05). This result is in line with Marchant et al. [69], who concluded after an in vitro assay that biofilm formation influences antibiotic resistance. But the presence of these resistance genes does not necessarily explain the resistance, as it can manifest itself through other mechanisms. However, in the literature, many authors have found no correlation between biofilm production and antibiotic resistance in *S. aureus*. Again, the discrepancies are explained by the variety of methods used to screen biofilm-producing strains [21]. To date, no indisputable conclusions have been proposed.

The genotypic evaluation of biofilm production in *Staphylococcus aureus* isolates showed that all the 8 genes encoding biofilm production (*fnbA, fnbB, icaA, icaB, icaC, icaD, clfA, clfB,* and *bap*) were detected in *Staphylococcus aureus* isolates. The attachment factor genes *icaA*, *icaB*, *icaC*, *icaD,* fnbA, and fnbB were present in all strong biofilm-producing strains, and a high number of these isolates (86.96%) carry *bap*, *cflA,* and *cflB*. Based on Fisher's exact test, there is a statistically significant relationship between the active genes and strong biofilm reaction (*p* value <0.05). Considering the high number of biofilm-producing strains, these results confirm that strains harbouring the i*caADBC* cluster [27], *clfB*, *fnbB, clfA*, and *fnbA* [70–72] are potential biofilm producers. In addition, the biofilm-associated protein (bap) plays an important role in the adherence of *S. aureus* and biofilm formation [73, 74].

## 5. Conclusions

In conclusion, this study revealed that there is a significant correlation between biofilm production and antibiotic resistance in *S. aureus* isolated from raw milk. A high number of multidrug-resistant strains carrying several biofilm-related genes were found. The presence of potentially biofilm-producing and antibiotic-resistant *S. aureus* in milk intended for human consumption represents a serious health hazard. These results indicate that the prevention and management of these infections should be a priority in Iran.

## Figures and Tables

**Table 1:** Oligonucleotide primers and PCR applications used to amplification of the biofilms genes encoding in *S. aureus* strains isolated from row cow milk.

Gene	Primer sequence (5′-3′)	PCR program	PCR condition	Size of product (bp)
*icaA*	F: GAC CTC GAA GTC AAT AGA GGT	1 cycle: 94°C—6 min. 33 cycle: 95°C—70 s 59°C—65 s 72°C—90 s 1 cycle: 72°C—8 min	5 *μ*l PCR buffer 10x 2.5 mm Mgcl_2_ 200 *μ*M dNTP (Fermentas) 0.5 *μ*m of each primers F&R 2 U Taq DNA polymerase (Fermentas) 3 *μ*l DNA template	814
	R: CCC AGT ATA ACG TTG GAT ACC			
*icaB*	F: ATC GCT TAA AGC ACA CGA CGC			526
	R:TAT CGG CAT CTG GTG TGA CAG			
*icaC*	F: ATA AAC TTG AAT TAG TGT ATT			989
	R: ATA TAT AAA ACT CTC TTA ACA			
*icaD*	F: AGG CAA TAT CCA ACG GTA A			371
	R: GTC ACG ACC TTT CTT ATA TT			

*fnbB*	F: ACGCTCAAGGCGACGGCAAAG	1 cycle: 95°C—4 min. 30 cycle: 95°C—45 s 58°C—60 s 72°C—40 s 1 cycle: 72°C—5 min	5 *μ*L PCR buffer 10x 2 mM Mgcl_2_ 200 *μ*M dNTP (Fermentas) 0.4 *μ*M of each primers F&R 1 U Taq DNA polymerase (Fermentas) 3 *μ*L DNA template	197
	R: ACCTTCTGCATGACCTTCTGCACCT			
*clfA*	F: CCGGATCCGTAGCTGCAGATGCACC			1000
	R: GCTCTAGATCACTCATCAGGTTGTTCAGG			

*Bap*	F: CCCTATATCGAAGGTGTAGAATTG	95°C—45 s 58°C—60 s 72°C—40 s 1 cycle: 72°C—5min	5 *μ*L PCR buffer 10x 2 mM Mgcl_2_ 200 *μ*M dNTP (Fermentas) 0.4 *μ*M of each primers F&R 1 U Taq DNA polymerase (Fermentas) 3 *μ*L DNA template	971
	R: GCTGTTGAAGTTAATACTGTACCTGC			
*fnbA*	F: ACGCTCAAGGCGACGGCAAAG			191
	R: ACCTTCTGCATGACCTTCTGCACCT			

*mecA*	F: AAAATCGATGGTAAAGGTTGGC	1 cycle: 94°C—5 min. 32 cycle: 94°C—60 s 55°C—60 s 72°C—2 min 1 cycle: 72°C—10 min	5 *μ*L PCR buffer 10x 2 mM Mgcl_2_ 200 *μ*M dNTP (Fermentas) 0.4 *μ*M of each primers F&R 1 U Taq DNA polymerase (Fermentas) 3 *μ*L DNA template	532
	R: AGTTCTGCAGTACCGGATTTGC			
*blaZ*	F: TACAACTGTAATATCGGAGGG			861
	R: CATTACTCTTGGCGGTTTC			
*vanA*	F: GGGAAAACGACAATTGC			732
	R: GTACAATGCGGCCGTTA			

*tet K*	F: GTAGCGACAATAGGTAATAGT	1 cycle: 95°C—5 min. 32 cycle: 94°C—60 s 59°C—60 s 72°C—2 min 1 cycle: 72°C—10 min	5 *μ*l PCR buffer 10X 2.5 mm Mgcl_2_ 200 *μ*M dNTP (Fermentas) 0.5 *μ*m of each primers F&R 2 U Taq DNA polymerase (Fermentas)	360
	R: GTAGTGACAATAAACCTCCTA			
*tet M*	F: AGTGGAGCGATTACAGAA			268
	R: CATATGTCCTGGCGTGTCTA			

*ermA*	F: AAGCGGTAAACCCCTCTGA	1 cycle: 94°C—6 min. 32 cycle: 94°C—60 s 57°C—60 s 72°C—2 min 1 cycle: 72°C—10 min	5 *μ*l PCR buffer 10x 2.5 mm Mgcl_2_ 200 *μ*M dNTP (Fermentas) 0.5 *μ*m of each primers F&R 2 U Taq DNA polymerase (Fermentas)	190
	R: TTCGCAAATCCCTTCTCAAC			
*ermC*	F: AATCGTCAATTCCTGCATGT			299
	R: TAATCGTGGAATACGGGTTTG			

*linA*	F: GGTGGCTGGGGGGTAGATGTATTAACTGG	1 cycle: 95°C—5 min. 32 cycle: 94°C—60 s 59°C—60 s 72°C—2 min 1 cycle: 72°C—10 min	5 *μ*L PCR buffer 10x 2 mM Mgcl_2_ 200 *μ*M dNTP (Fermentas) 0.4 *μ*M of each primers F&R 1 U Taq DNA polymerase (Fermentas) 3 *μ*L DNA template	323
	R: GCTTCTTTTGAAATACATGGTATTTTTCGA			

*aroA*	FA1: AAGGGCGAAATAGAAGTGCCGGGC	1 cycle: 95°C—5 min. 31 cycle: 94°C—60 s 62°C—60 s 72°C—2 min 1 cycle: 72°C—10 min	5 *μ*L PCR buffer 10x 2 mM Mgcl_2_ 200 *μ*M dNTP (Fermentas) 0.4 *μ*M of each primers F&R 1 U Taq DNA polymerase (Fermentas) 3 *μ*L DNA template	1153
	RA2: CACAAGCAACTGCAAGCAT			

**Table 2:** Distribution of biofilm-forming strains of *S. aureus* strains isolated from row cow milk.

Bacteria	Biofilm formation					
	Strong		Weak		Negative	
	NO	%	NO	%	NO	%
*S. aureus*	23	65.71	7	20	5	14.28

**Table 3:** Antibiotic resistance pattern in *S. aureus* strains isolated from row cow milk.

Antibiotic	Resistance	
	Number	%
Penicillin G	35	100
Ampicillin	32	91.42
Oxacillin	25	71.42
Methicillin	15	42.85
Kanamycin	15	42.85
Streptomycin	10	28.57
Tetracycline	10	28.57
Erythromycin	5	14.28
Lincomycin	5	14.28
Clindamycin	5	14.28
Gentamycin	2	5.71
Chloramphenicol	2	5.71
Vancomycin	0	0
Rifampicin	0	0

**Table 4:** Antibiotic resistance pattern based on biofilm reaction in *S. aureus* strains isolated from row cow milk.

Antibiotic	Strong biofilm producer				Weak biofilm producer				Negative			
	Resistance		Sensitive		Resistance		Sensitive		Resistance		Sensitive	
Penicillin G	23	100%	0	0%	3	42.85%	4	57.15%	2	40%	3	60%
Ampicillin	22	95.65%	1	4.35%	2	28.57%	5	71.83%	1	20%	4	80%
Oxacillin	22	95.65%	1	4.35%	2	28.57%	5	71.83%	2	40%	3	60%
Methicillin	15	65.22%	8	34.78%	2	28.57%	5	71.83%	2	40%	3	60%
Kanamycin	15	65.22%	8	34.78%	1	14.28%	6	85.71%	3	60%	2	40%
Tetracycline	9	39.13%	14	60.87%	2	28.57%	5	71.83%	1	20%	4	80%
Streptomycin	8	34.78%	15	65.22%	3	42.85%	4	57.15%	3	60%	2	40%
Erythromycin	5	21.74%	18	78.26%	4	57.15	3	42.85%	2	40%	3	60%
Lincomycin	5	21.74%	18	78.26%	4	57.15	3	42.85%	1	20%	4	80%
Clindamycin	4	17.39%	19	82.61%	3	42.85%	4	57.15%	3	60%	2	40%
Gentamycin	2	8.7%	21	91.3%	3	42.85%	4	57.15%	4	80%	1	20%
Chloramphenicol	2	8.7%	21	91.3%	3	42.85%	4	57.15%	1	20%	4	80%

**Table 5:** Prevalence of antibiotic resistance genes in *S. aureus* strains isolated from row cow milk.

Genes	blaZ	mecA	Aac-D	tetK	tetM	linA	ermA	ermB	linA
Positive *N*	25	15	15	10	10	5	5	5	5
%	71.42	42.85	42.85	28.57	28.57	14.28	14.28	14.28	14.28

**Table 6:** Prevalence of antibiotic resistance genes in biofilm-forming and nonforming in *S. aureus* strains isolated from row cow milk.

Antibiotic resistance genes	Biofilm reaction					
	Positive				Negative	
	Strong		Weak			
*blaZ*	23	100	4	57.14	1	20
*mecA*	15	65.22	4	57.14	1	20
*tet K*	10	43.48	3	42.85	0	0
*linA*	5	21.74	2	28.57	0	0
*tetM*	10	43.48	2	42.85	0	0
*erm A*	5	21.74	1	14.28	0	0
*ermB*	5	21.74	1	14.28	0	0
*Aac A-D*	15	65.22	5	71.43	1	20

**Table 7:** Genotypic evaluation of biofilm production in *S. aureus* strains isolated from row cow milk.

Genes	icaA	icaB	icaC	icaD	bap	fnbA	fnbB	clfA	clfB
Positive *N*	30	28	29	31	28	34	33	28	25
%	85.71	80	82.86	88.57	80	97.14	94.28	80	71.43

**Table 8:** Adherence of attachment factors in *S. aureus* strains isolated from row cow milk based on biofilm reaction.

Adherence genes	Biofilm reaction					
	Positive				Negative	
	Strong		Weak			
*ica A*	23	100	2	28.57	2	40
*ica B*	23	100	2	28.57	1	20
*ica C*	23	100	2	28.57	1	20
*ica D*	23	100	2	28.57	1	20
*Bap*	20	86.96	2	28.57	0	0
*fnbA*	23	100	2	28.57	0	0
*fnbB*	23	100	3	22.86	0	0
*clfA*	20	86.96	1	14.28	0	0
*clfB*	20	86.96	1	14.28	0	0

## Data Availability

The data supporting the findings of this article are available from the corresponding author upon request.
